# Safety perception in patients with advanced idiopathic Parkinson’s disease – a qualitative study

**DOI:** 10.3389/fnagi.2023.1200143

**Published:** 2023-09-06

**Authors:** Anna J. Pedrosa, Marlena van Munster, Lars Timmermann, David J. Pedrosa

**Affiliations:** ^1^Department of Neurology, University Hospital Giessen and Marburg, Marburg, Germany; ^2^Department of International Health, CAPHRI Care and Public Health Research Institute, Maastricht University, Maastricht, Netherlands; ^3^Centre for Mind, Brain and Behaviour, Philipps-University Marburg, Marburg, Germany

**Keywords:** Parkinson disease, patient safety, safety, quality of healthcare, palliative care, qualitative research

## Abstract

**Background:**

A fundamental cornerstone of quality of healthcare is patient safety, which many people with life-limiting illnesses feel is being compromised. Perceptions of impaired safety are associated with the occurrence of psychological distress and healthcare utilization. However, little is known about how people with idiopathic Parkinson’s disease (iPD) perceive their own safety toward the end of life. The aim of our study was therefore to investigate factors that influence the perception of safety of patients with advanced iPD.

**Methods:**

We conducted semi-structured interviews with a purposeful sample of 21 patients with advanced iPD. Participants were recruited at the neurology department of a tertiary care hospital in Germany between August 2021 and June 2022. Data were analyzed using reflexive thematic analysis.

**Results:**

iPD-patients reported relevant impairment of their safety. While most participants expressed safety concerns based on the manifestation of disease, our analysis identified enablers and barriers to establishing safety in patients with advanced iPD, in 10 additional domains: relationship to the disease, informedness, self-perception, utilization of support and care structures, healthcare professionals and structures, treatment, social interaction, social security, spirituality, and environment.

**Conclusion:**

This study provides new insights into safety perceptions of patients with advanced iPD, which extend well beyond the physical realm. The findings suggest that clinicians and policy makers should consider a holistic and multidisciplinary approach to assessing and improving patients’ safety taking into account the enablers and barriers identified in this study.

## Introduction

Idiopathic Parkinson’s disease (iPD) is a common neurodegenerative disorder that increases with age, and in its advanced stages, is characterized by impairment of activities of daily living, symptom burden, and reduced quality of life (Fasano et al., 2019; Feigin et al., 2019). Given the rapidly growing incidence (Feigin et al., 2019), it is a logical consequence that the disease has been recently declared a public health issue with the aim of improving the care of iPD-patients (World Health Organization, 2022). This seems all the more important as iPD specialists are not confident about the quality of care their patients receive in hospital, leading to the recommendation that hospital staff and clinicians should be thoroughly educated about the disease, including management, potential complications and medications to avoid (Chou et al., 2011).

This knowledge contributes to the understanding that one of the pillars of high-quality healthcare is patient safety (World Health Organization, 2019). According to a white paper, patient safety is defined as the extent, determined from the patient’s perspective, to which individuals, professional groups, teams, organizations, associations and the healthcare system

1. are in a state where adverse events are rare, safety behaviors are promoted and risks are controlled,
2. are able to recognize safety as a worthwhile goal and implement realistic options for improvement, and
3. are able to use their innovation skills to achieve safety (Schrappe, 2018).

Consequently, patients are assigned an essential role in defining and advancing patient safety (Schrappe, 2018; World Health Organization, 2021). Patients are thought to provide valuable insights into safety that complement existing measurement of patient safety and challenge common definitions of patient safety incidents that focus on physical risks (O’Hara et al., 2018). In this context, although there is a large number of studies dealing with safety aspects in patients with advanced iPD, they are mostly limited to side effects of available treatment options. However, evidence on patient safety in mixed populations with life-limiting conditions suggests that a patient-centered approach must consider safety issues beyond the physical domain to meet patient priorities in healthcare (Pedrosa Carrasco et al., 2021, 2022). These may include factors (e.g., risk behaviors in informal care, abuse, unsafe neighborhoods, natural disasters) that are not a function of healthcare itself, but which may be essential to consider in order to provide holistic care. This is all the more important given that a perception of compromised safety among patients is not only associated with pronounced psychological symptoms such as anxiety and depression (Vincent and Coulter, 2002), but in populations of severely ill patients has been shown to make the decision to present to hospital more likely (Henson et al., 2016; Robinson et al., 2018).

Nevertheless, despite the fundamental importance of patients themselves in establishing safety in the healthcare system, little is known about iPD-patients’ own perceptions of safety. A comprehensive understanding of this could yet be used to promote a differentiated and individualized approach to both safety assessment and the implementation of safety measures. This study set out to examine experiences of hospital patients with advanced iPD to ultimately gain knowledge on how to improve patient safety and, therefore, care for iPD-patients across the care continuum.

## Methods

### Study design

We conducted a qualitative study to explore the experiences and views on perceived safety in order to identify enablers and barriers. The Consolidated Criteria for Reporting Qualitative Research (COREQ) guided reporting (Tong et al., 2007).

### Recruitment

We recruited iPD-patients with advanced disease according to current literature (Antonini et al., 2018) under the care of the Department of Neurology at the University Hospital of Marburg, Germany. Participants were purposefully selected according to key characteristics (age, gender, marital status, living situation and history of migration) (Bowling, 2014) to capture a breadth of views and ensure diversity. Patients were not deemed eligible if they had insufficient German language skills or cognitive inability to give informed consent. Eligibility was determined by the treating physician, but patients were invited to participate by the interviewer.

### Data collection

Participants’ socio-demographic data were collected prior to the interview. The research team with expertise in neurology, palliative care and medical ethics developed an interview guide for semi-structured qualitative interviews. The guide comprised open-ended questions aiming at the participants’ experiences and perceptions of safety (*cf.* Supplementary Table 1 for English translation). All interviews were conducted face-to-face on the hospital premises by AP. In some cases, due to the immobility of the participants, it was necessary to conduct the interview in the patient’s room. However, privacy was always ensured. There was no pre-existing relationship between participants and interviewer. Interviews were digitally audiotaped and transcribed verbatim. Care was taken to anonymize any identifiable references to participants, staff or care providers. For publication, illustrative quotes were pseudonymized and translated from German into English by a fluent speaker. The sample size allowed for an in-depth exploration of an under-researched aspect and provided rich data appropriate to answer the research question (Braun and Clarke, 2021b).

### Analysis

MAXQDA was used to assist in data management. We performed reflexive thematic analysis following six stages: (1) familiarization with data, (2) development and refinement of inductive codes, (3) constructing of initial themes, (4) development and review potential themes (5) refinement, definition, and naming themes, (6) reporting (Braun and Clarke, 2021a). For the first five interviews, AP and MvM independently created codes from which a coding scheme was reached through consensus discussions. The resulting preliminary coding scheme was applied by AP to the analysis of the remaining interview transcripts and expanded as new codes and themes were identified.

## Results

### Participants

We approached 22 iPD-patients of whom 21 consented to participate. Interviews were performed between August 2021 and June 2022 and had a median length of 20 min (range 8–59 min). One interview had to be terminated early because the participant suddenly felt too unwell to be able to concentrate sufficiently on the questions being asked. However, the data already collected could be used for our analysis. Detailed participant characteristics are shown in Table 1. Supplementary Table 2 shows the percentage distribution of the individual indicators of advanced iPD identified by Antonini et al. (2018).

**Table 1:** Participant characteristics.

Demographics	
Gender (%)	
Female	11 (52.4)
Male	10 (47.6)
Median age in years (range)	66 (49–84)
Median time since diagnosis in years (range)	9 (1–30)
Civil status (%)	
Married	15 (71.4)
Single	2 (9.5)
Divorced	3 (14.3)
Widowed	1 (4.8)
First-generation immigrants and/or holders of dual citizenship (%)	5 (23.8)
Professional education (%)	
None	3 (14.3)
Non-university degree	11 (52.4)
University degree (including technical college)	7 (33.3)
Living situation (%)	
Own household	17 (81.0)
With relatives	1 (4.8)
Assisted living	1 (4.8)
Nursing home	2 (9.5)
Clinical context (%)	
Inpatient Parkinson complex treatment	15 (71.4)
Emergency hospitalization	2 (9.5)
DBS implantation	1 (4.8)
Medication pump installation	1 (4.8)
3-month follow-up after DBS implantation	1 (4.8)
Routine outpatient visit	1 (4.8)

DBS, deep brain stimulation.

### Main findings

**Figure 1:**
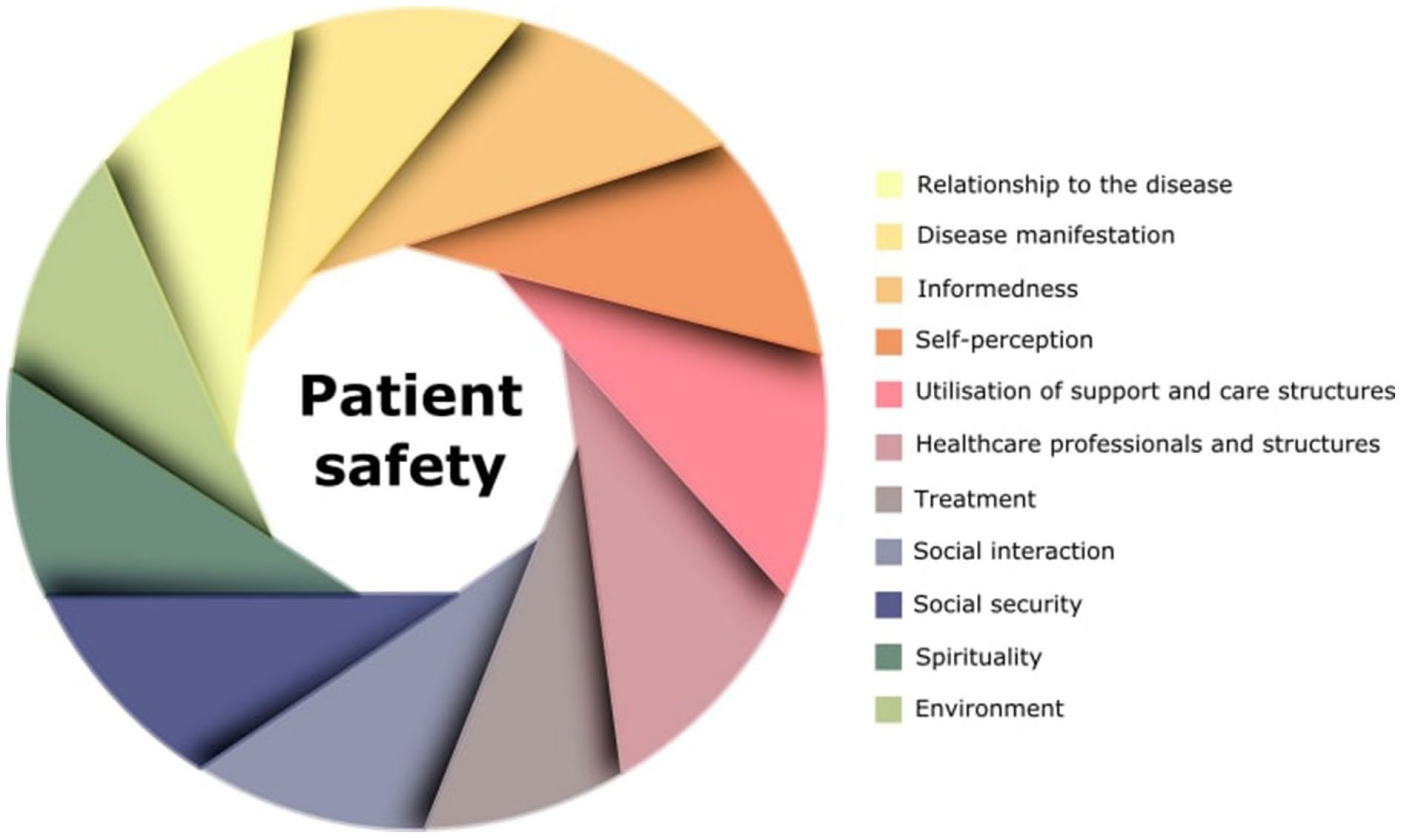
Domains relevant to patient safety in iPD.

We identified barriers and enablers to the implementation of patient safety in iPD-patients. These were related to 11 themes, which are presented in Table 2. Figure 1 shows the multidimensional architecture of patient safety in iPD based on the identified themes.

**Table 2:** Themes and subthemes relevant to iPD-patients’ perception of safety divided into enablers and barriers.

Themes	Enablers	Barriers
Relationship to the disease	Coping	
	Openness	
Disease manifestation		Motor symptoms
		Non-motor symptoms
		Comorbidities
		Uncertain future
Informedness	Own informedness	Lack of information
	Informedness of relatives	
Self-perception	Activity	Everyday dysfunctionality
		Anxiousness
Utilization of support and care structures	Healthcare facilities and services	
	Residential care and assisted living	
	Informal support services	
Healthcare professionals and structures	Professional Expertise	Lack of professional expertise
	Trust in healthcare professionals	Lack of trust in healthcare professionals
	Patient-centered care	Deficits in professional-patient communication
		Structural deficits of healthcare
Treatment	Non-medical treatment	Complications and side effects of medication
	Medical treatment	DBS-Implantation
	Medical aids	
Social interaction	Support from family and friends	Weaknesses in the family structure: Lack of support Family’s breaking point Difficulties in living up to the partnership Family conflicts
	Thoughtfulness of others	Hurdles in social life: Lack of understanding and stigmatization Social overstrain Social isolation
Social security	Professional stability	Incapacity to work and job loss
	Financial stability	Financial stress
Spirituality	Drawing from faith	
Environment	Familiar environment	Unfamiliar environment
	Barrier-free living	Physical barriers
	Routine	Discontinuity in routine
		Traffic
		COVID pandemic

### Relationship to the disease

The relationship to their illness in terms of processing and acceptance was crucial for the participants’ perceptions of safety. In particular, coping with the disease itself was associated with an increased sense of safety. This was fostered, among other factors, by optimism, resilience and empowerment through achievement.

*“I didn't let the illness affect me and I didn't hang my head, in that sense. It was like this: I had the disease in a pocket. It was there, but I still lived normally.” (Participant 2, male, 63 years)*

For some, safety meant being open about the disease and its associated symptoms, as hiding the condition from others was associated with fear of discovery.

*“I always say that if you are open about it and if you also say: Hey, I'm not feeling so well today, that's easier than retreating into a quiet closet.” (Participant 11, female, 53 years)*

### Disease manifestation

Almost all participants agreed on feelings of insecurity caused by symptoms of the disease. This was mainly the case for motor symptoms, where actual falls or perceived risk of falls were particularly emphasized.

*“I've already fallen a few times. Sometimes I feel a bit tingly. And my hands, I already have so much pain there, and my arms, I feel unsafe. Not as safe as it should be.” (Participant 16, male 76 years)*

However, participants also attributed special relevance to non-motor symptoms in the experience of safety such as pain, constipation and incontinence. However, physical safety was rarely mentioned in this context; the safety affected by these symptoms was mainly deemed to be of social nature, which for some encouraged social withdrawal.

*“When you go travelling...that's not possible, if only because of the incontinence. I have pads that can absorb most of it. But you can see that the clothes, that it shows through.” (Participant 15, male, 72 years)*

Furthermore, some participants reported psychological safety concerns due to symptoms such as depression, panic attacks and delusions. For two participants, the psychological distress even culminated in suicidal thoughts, with one of them being admitted to a psychiatric hospital.

*“Because of these panic attacks that build up, I always think about suicide or 'Is my wife doing what we agreed in the advance directive? Will she make sure that I have a proper death?” (Participant 12, male, 61 years)*

Uncertainties regarding the future were another source of feeling unsafe. This mainly concerned fears about the course of the chronic progressive disease including the end of life, the associated need for care and unpleasant connotations with dependence.

*“Of course, I’m thinking about the future and how it will go on […] Those are the two insecurities. So one is experienced insecurities (.) and the other is fear of the future.” (Participant 3, male, 67 years)*

### Informedness

Being informed about Parkinson’s disease, but also having well-informed relatives, was perceived by many as promoting safety as it resulted in mental preparation and empowerment.

*“Yes, it is definitely important […] to say that this is the reason why this is the way it is, this is how it comes about. It's always better to be in the know, because if you only find out about it today or live according to it, you have been given a lot of quality of life.” (Participant 2, male, 63 years)*

Conversely, two participants reported that a lack of information about the disease and its treatment led to a relevant feeling of insecurity.

*“When you are given medication that you don't know, it makes you insecure as to how it is compatible with all the other medication that I have to take every day. I would have hoped for a bit more information, but you don't get a proper answer anywhere.” (Participant 21, female, 75 years)*

### Self-perception

Participants also reflected on their own nature and role as actors in creating safety or insecurity. Some expressed safety concerns in their daily lives because they found themselves overwhelmed or uncomfortable being dependent on others. Most participants experienced a sense of safety as long as they were physically active as they felt more mobile and were less aware of the physical symptoms.

*“I usually feel safer when I move properly. When I walk, when I pace, I generally feel safer because then I feel active and I think to myself: 'Parkinson's is actually nothing. It can't affect me'.” (Participant 13, male, 49 years)*

However, one participant also stated that she had felt anxious and insecure all her life due to her personality traits, which provided a fragile foundation for her patient safety in the context of iPD.

*“Yes, it’s because I’ve always been afraid all my life, although it’s not a thing to be afraid of. I’m always afraid of Santa Claus in disguise, when I see his face.” (Participant 6, female, 78 years)*

### Utilization of support and care structures

Utilization of support and care structures was perceived valuable in providing safety. This included healthcare facilities and services, residential care and assisted living as well as informal support services such as support groups and volunteers.

*“So what gives me safety now is that I’m in a nursing home. I find that quite safe.” (Participant 6)*

### Healthcare professionals and structures

Interactions with healthcare professional also played a major role in participants’ perceived safety. Professional expertise and, closely related to this, the trust placed in staff had a positive effect. In addition, however, patient-centered care encompassing empathy, dedication of time and holistic approaches was considered vital for the experience of safety.

*Participant: “Yes, they [care team] gave me safety.”*

*Interviewer: “What was decisive for that?”*

*Participant: “Humanity, ability, knowledge. (…) Yes, competence and humanity.” (Participant 18, female, 54)*

However, when these characteristics were absent in the staff-patient-relationship, patient safety could be compromised. In particular, many participants reported communication deficits, a lack of compassion and a feeling of not being heard. For example, participants reported that healthcare professionals - unaware of the fluctuating symptoms associated with the on–off phenomenon - blamed participants for being lazy when in fact they were suddenly unable to perform certain activities.

*“I rang the bell and said: “Can you please help me go to the toilet?” She hung her hands and said: “No, you can go yourself! I can’t help you! You walk during the day and you can’t walk at night.” And I said: “Yes, if I can, I’ll do it myself”. But I couldn’t go.” (Participant 9, female, 76 years)*

But structural deficits in care, such as work overload, lack of communication between professional groups or discontinuity in care were also among risk factors for reduced sense of safety.

*“But he [former treating physician] is no longer at the hospital and since he left, I have had different doctors all the time […] Everyone always tries their best and messes around [with DBS]. And that always worse.” (Participant 11, female, 53 years)*

### Treatment

The therapeutic success of the different non-medical and medical treatment methods alone brought a sense of security for many participants.

*On the second day after [DBS-]surgery, I danced with my friends in the hospital room. […] This experience that they were able to help me so well with this is the best thing I have against my fears. (Participant 3, male, 67 years)*

However, feared and experienced complications and side effects of the various therapy options could also be a reason for feeling unsafe.

*Every new [medication] adjustment is associated with a change and this usually makes you feel insecure. (Participant 1, female, 52 years)*

Mobility-enhancing aids, especially walkers, were seen by many participants as a way of improving safety in the face of the risk of falling.

*“I’m safer with the walker. I’m safer with it in my hand than without it. There’s no doubt about that. Also with the wheelchair, if things are not going well you can sit in it. You’re also better off with it.” (Participant 5, male, 58 years)*

### Social interaction

Support of a practical and emotional nature from the personal environment of the participants, especially the family, was perceived as promoting safety.

*“When the partner is there! That’s very safe then! Or if siblings, close relatives or people who are close to me. I’m safe then.” (Participant 19, female, 64 years)*

Yet, weaknesses noticed in the family structure could contribute to a feeling of insecurity: lack of support, overburdened family carers, patient’s struggle to living up to the partnership, and family conflicts.

*“So I’m afraid (…) that I won’t be able to maintain the minimum with my wife, because I live with her. I am also afraid of that. I don’t think she would leave me, but (…).” (Participant 3, male, 67 years)*

While an understanding attitude of others toward the person with iPD was helpful, the participants often felt insecure due to shortcomings in social life. In addition to stigmatization, they frequently experienced the feeling of being overwhelmed when surrounded by other people.

*“We just choose what I can eat. A bread dumbling, my husband cuts it, he then cuts it into small pieces and I can eat it with a spoon. But that is difficult. At the beginning I thought: ‘Uh, now everyone is looking. The world is over.” (Participant 19, female, 64 years)*

Participants regretted that they felt alone or socially withdrawn, which had a negative impact on their sense of security.

*“You feel alone and abandoned. Before I couldn’t – I was willing to talk to some people. I don’t do that anymore. That questioning, that – I don’t know, I have a feeling of being unsafe already. But I don’t know what to do about it.” (Participant 10, female, 84 years)*

### Social security

Some participants mentioned that social security through professional and financial stability gave them a feeling of safety as they believed to be prepared for the contingencies of an uncertain future.

*“If you have enough dough [money], it’s no problem, but that’s the prerequisite.” (Participant 14, male, 79 years)*

Others, however, saw their safety compromised in this respect due to the illness, complained about financial disadvantages as a result of being unable to work, taking early retirement or losing their job.

*“Because my job is dangerous, a crane driver. For example, I worked at the top in the cabin and downstairs there were 60, 80 people working at the steelworks and they said: ‘You can’t come in here, you’re not allowed to work.’ Well, the factory doctor said I ought to retire.” (Participant 17, male, 66 years)*

Two female participants even reported financial losses due to a gambling addiction most likely caused by the medication.

*“Yes, lots and lots of side effects. For example, shopping. I used to go shopping, I had everything at home, but still I went shopping at the shop. I always wanted to go to the casino. When I was in [city] in [country] I went to the casino twice a week. And I spent a lot of money.” (Participant 9, female, 76 years)*

### Spirituality

No participants reported spiritual or religious challenges that affected their sense of safety. However, a few participants felt that faith supported their sense of safety in the experience of illness.

*“People need something to hold on to. Yes, even if you say that not everything can be like that, but I think there is a point, how should I put it, where you can hold on to it.” (Participant 21, female, 75 years)*

### Environment

Environmental factors played a vital role in safety establishment. Thus, physical barriers in walking routes in the home and the public were perceived as a relevant threat to safety mostly due to increasing the risk of falls.

*“I have changed flats. Before, I had to go down steep stairs. Then I gave that up because there was too much danger of falling down the stairs and so on.” (Participant 15, male, 72 years)*

iPD-patients felt more comfortable in familiar surroundings whereas unknown environments created a sense of insecurity. In addition, routine in daily life beyond regular medication was given special importance whereas discontinuation of routine was perceived as a safety barrier.

*“Yes, I think that [routine in nursing home] is safety for me. You always have everything on time at the same time and so on. I think so.” (Participant 6, female, 78 years).*

Participation in road traffic, especially as a car driver, but also as a pedestrian, was perceived as a safety hazard by some iPD-patients interviewed.

*“Until last year, I went to visit the children. I went by car, but that's no longer possible, they have to come to me. I don't like risk, I don't like it. It's a risk when I drive a car, if I'm not well adjusted with medication, the day doesn't go well.” (Participant 8, female, 59 years)*

Additionally, current world events such as the COVID pandemic were seen as a barrier to an intact sense of safety, as there were fears of an unfavorable course of infection.

*“Yes, it was all very scary. Although I was one of the first ones to go with this [...] AstraZeneca. And I was actually vaccinated so early and had to wait three months and that made me feel unsafe.” (Participant 5, male, 58 years)*

## Discussion

Two major conclusions can be drawn from our qualitative study: (i) the perception of safety in advanced iPD corresponds to a multidimensional construct, and based on this, (ii) a holistic and multi-professional approach in iPD care is indisputable to ensure safety.

Advanced iPD is associated with higher disability and mortality (Barer et al., 2022). Our research indicates that patients’ safety perception in advanced stages of the disease is compromised and confirms previous assumptions that patient safety toward the end of life is shaped by the care provided by healthcare staff and informal caregivers, but also by patient-related factors and external factors (Pedrosa Carrasco et al., 2021). We identified various enablers and barriers to patients’ perceptions of safety at physical, psychological, social, and spiritual levels.

Participants shared challenges in engendering safety, as well as safety-giving measures during their journey through the healthcare system. Undoubtedly, our study revealed that motor and non-motor symptoms associated with iPD, as well as uncertainty and worries about the future course of the degenerative disease, can lead to feelings of insecurity. It is not surprising, therefore, that therapeutic efforts were considered to be of great importance for the generation of safety. In this context, however, thorough education about medication, including potential side effects, could enhance a sense of safety and promote patient empowerment. That being said, healthcare which deviates from patient needs or grievances have led to a loss of safety among iPD-patients (Owen et al., 2022). This phenomenon does not seem to be unfounded, as iPD-patients are prone to inadequate prescribing and complications during hospitalizations (Gerlach et al., 2011; Mantri et al., 2019; Bakker et al., 2022; Richard et al., 2022). In our study, participants frequently described insufficient understanding of the disease and lack of expertise in terms of treatment specifics on the part of healthcare professionals as the cause of feeling unsafe. This underscores the usefulness of early collaboration among disciplines and facilitated short-term access to centers with movement disorder expertise for iPD-patients with specialized therapeutic approaches. In addition, in the community specially trained nurses could provide care coordination, patient navigation, and information to complement existing care structures (van Munster et al., 2022). Palliative care services may offer further support to promote safe care for patients with advanced iPD (Fleisher et al., 2020).

But even as private individuals outside the healthcare system, people with iPD often felt their safety threatened when they were exposed to social challenges in society. Even though social safety schemas are assumed to develop early in life, these perceptions are shaped by the actual situations people face (Slavich, 2020). In this respect, our study reflected that social self-schemas and world-schemas were of prominent importance in the experience of safety of people with iPD. Social overload and experienced or perceived stigmatization as a result of their condition were described as relevant safety barriers, leading to social withdrawal in many patients. In our study, these social safety schemas were closely interwoven with psychological aspects of safety. Psychological safety concerns result in a reduction in feeling comfortable being and expressing oneself, and sharing worries and failures without fear of embarrassment, ridicule, shame or retribution (Torralba et al., 2020). The study participants reported impaired general social functioning, but also narrated more specific insecurities in the family environment. For example, in line with earlier research revealing negative effects on couple and family relationships (Vatter et al., 2018; Perepezko et al., 2019), some participants feared being a burden to their family, reported disputes grounded in the disease and their insecurity about not being able to fulfil their partnership in the future.

In a North American study, higher spirituality was associated with less impairment of quality of life and less anxiety and depression in patients with Parkinson syndromes, suggesting a potential influence on feelings of safety (Prizer et al., 2020). Given that a minority of Germans perceive themselves as particularly spiritual or religious (Statista, 2017; YouGov, 2021), it was, however, not unusual that none of the participants reported spiritual or religious crises that led to feelings of insecurity. Nevertheless, for a few iPD-patients interviewed, faith and the idea of a divine plan gave them a sense of safety which might be explained by a protective effect against psychological distress (Bernard et al., 2017). Other research projects could revisit the influence on perceived safety in populations where religion and spirituality are more prominent in everyday life.

In summary, while the identified enablers and barriers to the safety of iPD-patients should be considered to ensure a holistic approach to care, our study also provides direct recommendations for healthcare professionals to promote patient safety. These include a sound knowledge of the disease and its treatment options, prescription of medical aids, as well as soft skills such as empathy, patient-centered communication, and teamwork. The basic prerequisite for understanding individual patient safety is, therefore, a thorough medical history that goes beyond the patient’s physical complaints to address unmet needs.

## Strength and limitations

Our qualitative approach supported explorative investigation of a variety of aspects that contribute to and hinder perceptions of safety in patients with advanced iPD. Nevertheless, there are some important limitations. Recruitment under heterogeneous clinical conditions and exclusion of patients with advanced iPD with severe cognitive impairment may have introduced selection bias. Moreover, given that recruitment took place at a single site in Germany, views may not be representative of patients from other geographical regions. Further research outside Germany could identify country- and health system-specific characteristics of patient safety. In addition, our interview study provided a snapshot; a longitudinal study might yield more information about the variability or persistence of feelings of insecurity.

## Conclusion

Our study shows that the perception of safety of people with iPD is subject to a holistic concept based on physical, psychological, social, and spiritual needs. In order to identify safety concerns and address safety issues, multidisciplinary approaches to the care of people with iPD should be adopted. This may include systematically assessing safety concerns and developing safety strategies for patients, taking into account individual characteristics and circumstances. The enablers and barriers identified by our research offer promising starting points for improving the quality of healthcare by informing safety-oriented practice. Nevertheless, complementary quantitative research projects are warranted to estimate the prevalence of individual safety concerns and thus their implications for the healthcare system, and to inform quality improvement initiatives by healthcare professionals and policy makers.

## Data availability statement

The raw data supporting the conclusions of this article will be made available by the authors, without undue reservation.

## Ethics statement

The studies involving humans were approved by Ethics Committee of the Medical Faculty of the Philipps University of Marburg. The studies were conducted in accordance with the local legislation and institutional requirements. The participants provided their written informed consent to participate in this study.

## Author contributions

AP and DP conceptualized and planned the study. AP conducted the interviews and drafted the manuscript. AP and MvM were involved in the qualitative analysis of data. All authors provided critical comments on drafts of the manuscript and approved the final manuscript.

## Funding

AP received funding through the Support Fund for Female Doctoral Candidates and Postdocs with Family Responsibilities of the Philipps-University Marburg. Open Access funding provided by the Open Acess Publishing Fund of Philipps-Universität Marburg with support of the Deutsche Forschungsgemeinschaft (DFG, German Research Foundation).

## Conflict of interest

DP received payments as a consultant for Boston Scientific and as a speaker at symposia sponsored by Boston Scientific, Zambon and AbbVie. DP’s institution, not DP personally, received funding from the German Federal Joint Committee (G-BA), the German Federal Ministry of Education and Research, the Horizon 2020 program of the European Commission, Boston Scientific, the Parkinson’s Foundation, the Dr. Reinfried Pohl Foundation, and the German Parkinson Association (dPV). LT reports grants, personal fees and non-financial support from Medtronic, Boston Scientific, St. Jude Medical, UCB Schwarz Pharma, Archimedes Pharma, TEVA Pharma, Lundbeck Pharma, Desitin Pharma, Abbvie; personal fees and non-financial support from SAPIENS; grants and personal fees from Bayer Healthcare; personal fees from Medas Pharma, TAD Pharma; personal fees and non-financial support from GlaxoSmithKline, Orion Pharma; grants from Zur Rose Pharma, outside the submitted work.

The remaining authors declare that the research was conducted in the absence of any commercial or financial relationships that could be construed as a potential conflict of interest.

## Publisher’s note

All claims expressed in this article are solely those of the authors and do not necessarily represent those of their affiliated organizations, or those of the publisher, the editors and the reviewers. Any product that may be evaluated in this article, or claim that may be made by its manufacturer, is not guaranteed or endorsed by the publisher.
