# Supplementary material for: Safety perception in patients with advanced idiopathic Parkinson’s disease – a qualitative study
Source: Front Aging Neurosci. 2023 Sep 6;15:1200143. doi: 10.3389/fnagi.2023.1200143 (PMC10513089; doi:10.3389/fnagi.2023.1200143)
Supplement: Supplementary file 1 [file Table_1.DOCX]

**Interview guide**

| **Questions** | **Additional questions if topic not covered** |
| --- | --- |
| You suffer from Parkinson's disease, has the disease or its effects ever made you feel unsafe? | Have you ever felt unsafe (apart from physical effects) during the course of the disease because of emotional or psychological problems? |
| From your point of view, what promotes the feeling of insecurity in patients with Parkinson's disease? | Have you ever felt unsafe (apart from physical effects) during the course of the disease because of spiritual or religious problems? |
| What, on the other hand, promotes a sense of security in patients with Parkinson's disease? | Have you ever felt unsafe (apart from physical effects) during the course of the disease because of social problems? |
| Did you feel so unsafe or threatened at any time in the past because of your disease so that you had to seek help from relatives or friends? | Looking back, has treatment for Parkinson’s disease ever made you feel unsafe? |
| Did you feel so unsafe or threatened at any time in the past because of your illness so that you had to seek professional help, e.g. from the nursing service, doctors or psychologists? | Looking back, has your care team's interaction with you ever made you feel unsafe? |
| Can you think of aids or measures that your care team can take to increase Parkinson's patients' sense of safety? | Looking back, has your personal environment's interaction with you ever made you feel unsafe? |
