# Supplementary material for: Safety perception in patients with advanced idiopathic Parkinson’s disease – a qualitative study
Source: Front Aging Neurosci. 2023 Sep 6;15:1200143. doi: 10.3389/fnagi.2023.1200143 (PMC10513089; doi:10.3389/fnagi.2023.1200143)
Supplement: Supplementary file 2 [file Table_2.docx]

Study participants met indicators of advanced Parkinson's disease

| Criteria | n (%) |
| --- | --- |
| Motor symptoms |  |
| Moderate level of troublesome motor fluctuations | 11 (52.4) |
| At least 2 h of the waking day with “off” symptoms | 11 (52.4) |
| At least 1 h of the day with troublesome dyskinesia | 3 (14.3) |
| Moderate level of dyskinesia | 2 (9.5) |
| Troublesome dysphagia | 2 (9.5) |
| Daily oral levodopa doses “At least 5 times a day” | 13 (61.9) |
| Non-motor symptoms |  |
| Mild level of dementia | 0 (0.0) |
| Non-transitory troublesome hallucinations | 5 (23.8) |
| Moderate level of psychosis | 0 (0.0) |
| NMS fluctuations | 9 (42.6) |
| Moderate level of nighttime sleep disturbances | 6 (28.6) |
| Functional impacts |  |
| Repeated falls despite optimal treatment | 6 (28.6) |
| Needs help with ADLs at least some of the time | 10 (47.6) |
| Not able to perform complex tasks at least some of the time | 11 (52.4) |
| Moderate impaired mobility | 17 (81.0) |
